# A novel alternative to suture passer for closure of fascial defect in laparoscopic ventral hernia repair. A case report

**DOI:** 10.1016/j.ijscr.2019.06.045

**Published:** 2019-06-26

**Authors:** Jun Han Lai, Guo Hou Loo, Mohamad Aznan Bin Shuhaili, Nik Ritza Kosai

**Affiliations:** Department of General Surgery, Faculty of Medicine, The National University of Malaysia, Jalan Yaacob Latiff, Bandar Tun Razak, Postcode 56000, Selangor, Malaysia

**Keywords:** Laparoscopic IPOM plus, Transfascial suture passer, Incisional hernia, Mesh hernia repair, Bridged repair

## Abstract

**Introduction:**

Primary fascial closure can be a challenging step during a laparoscopic intraperitoneal onlay mesh (IPOM) repair for a ventral hernia.

**Case presentation:**

We present here a novel technique of using intravenous (IV) cannula as an alternative to suture passer for fascial closure during laparoscopic IPOM repair for a 59-year-old patient with an incisional ventral hernia. The placement of non-absorbable sutures for fascial closure was done with the help of a 14 gauge IV cannula instead of a transfascial suture passer. The rest of the procedural steps were the same as a standard laparoscopic IPOM repair. The patient's post-operative recovery was uneventful.

**Discussion:**

Primary fascial closure during a laparoscopic IPOM hernia repair can be done either by intracorporeal or extracorporeal techniques, using interrupted or continuous sutures. We propose a novel alternative to suture passer in primary fascial closure. IV cannulas are widely available in hospital settings. The advantage of using an IV cannula instead of a suture passer is that they are widely available. Its single-use also eliminates the risk of transmissible diseases, and as it has a smaller diameter than suture passer, it requires a lower insertion force for successful placement.

**Conclusion:**

An IV cannula may be used as a more economical alternative to a transfascial suture passer. This technique is easily reproducible and does not violate the principles of primary fascial defect closure in laparoscopic ventral hernia repair.

## Highlights

• Transfascial suture passer has been traditionally used to perform primary fascial closure in laparoscopic IPOM hernia repair.
• We propose a novel alternative to suture passer in primary fascial closure by using large-bore intravenous (IV) cannula.
• The advantage of using IV cannula is that it is widely available and requires a lower insertion force.
• This is the first published report on using IV cannula as an alternative to suture passer for fascial closure in laparoscopic IPOM repair.
• This technique is easily reproducible and does not violate the principles of primary fascial defect closure.

## 1 Introduction

Ventral hernia repair a bread and butter surgery, can be a great challenge to surgeons. Laparoscopic repair seems to be better than open repair in the short term, where it conveys a shortened hospital stay, reduced surgical site and mesh infection, reduced postoperative pain and faster recovery. The need for fascial defect closure in laparoscopic intraperitoneal onlay mesh (IPOM) hernia repair continues to be debated. There is evidence to suggest the benefits of primary fascial closure during laparoscopic IPOM repair such as reduced seroma formation. Transfascial suture passer has been traditionally used to perform primary fascial closure. Here, we propose a novel alternative to suture passer in primary fascial closure by using large-bore intravenous (IV) cannula. The advantage of using IV cannula is that it is widely available, requires a lower insertion force for successful placement, and less risk of transmissible diseases. This is the first published case report on using IV cannula as an alternative to suture passer for primary fascial closure in laparoscopic IPOM repair. This work has been reported in line with the SCARE criteria [13].

## 2 Case presentation

**Fig. 1:**
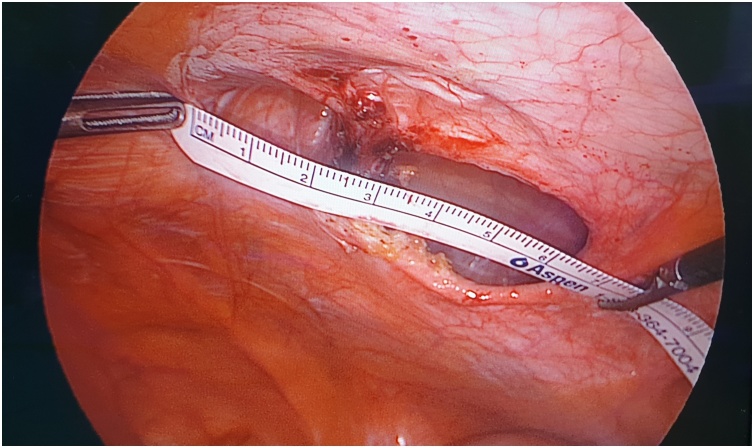
Laparoscopic view intraoperatively showing fascial defect which measures 6 cm in the largest dimension.

A 59-year-old lady with a BMI of 27 kg/m^2^ and no comorbid underwent an open appendicectomy via a Lanz incision for perforated appendicitis. There was a superficial surgical site infection which was treated by dressing followed by secondary suturing. Three years later, she presented to us with an incarcerated incisional hernia. We performed a laparoscopic intraperitoneal onlay mesh (IPOM) repair for her. Intraoperatively, standard port placement was done, followed by adhesiolysis of small bowel segments from the hernia sac. The fascial defect measures 6 cm in the largest dimension (Fig. 1). Prior to the mesh fixation, primary fascial closure was done using non-absorbable sutures (Prolene^®^ 0) passed extra-corporeally with the help of an intravenous cannula BD Angiocath™ (14 gauge) instead of a transfascial suture passer. The rest of the procedural steps were the same as a standard laparoscopic IPOM repair. Post-operative recovery was uneventful, and during her follow-up six months later, she has no hernia recurrence or chronic pain.

## 3 Discussion

Up to 20% of patients develop incisional hernias after a midline laparotomy, and the incidence is doubled if the index operation is complicated by surgical site infection [1]. This bread and butter surgery continues to be a great challenge to surgeons. The recurrence rate following repair of ventral incisional hernia is much higher than primary ventral hernia and may reach up to 43% at three years [2]. Studies seem to suggest that laparoscopic repair is advantageous in the short-term as compared to open repair as it offers shortened hospital stay, reduced surgical site and mesh infection, reduced postoperative pain and faster recovery [3,4]. Some authors suggest that laparoscopic repair is preferred in ventral hernia with defects between 4 cm–10 cm in a clean field [5].

The need for primary fascial closure in laparoscopic IPOM repair continues to be debated. A meta-analysis and one prospective trial indicate that primary fascial closure is associated with fewer adverse hernia site events, i.e. reduced seroma formation and a shorter hospital stay [6]. Another systematic review which included 11 studies showed that primary fascial closure is associated with lower recurrence rates, reduced clinical bulging and seroma formation. Patients with closure were also more satisfied and had a better functional outcome [7]. However, two large retrospective studies showed that primary fascial closure was not associated with reduced hernia recurrence, seroma formation or surgical site infection [8,9]. Given the lack of high-quality data to guide practice, some authors advocate primary fascial closure in hernia defects more than 3 cm width [5].

Primary fascial closure during a laparoscopic IPOM hernia repair can be done either by intracorporeal or extracorporeal techniques, using interrupted or continuous sutures [10]. Many techniques of fascial closure have been described, but the simplest closure method is the extracorporeal interrupted suture technique described by Franklin et al. [11]. A thick non-absorbable suture of size 0–2 is usually preferred in fascial closure [10]. A 1 cm interval between sutures is recommended, except in the ‘shoelacing’ technique, where a 3 cm interval is acceptable [10].

Transfascial suture passer has been traditionally used to perform primary fascial closure [12]. There are many types of commercially available suture passers. In our centre, we routinely use a reusable transfascial suture passer (BERCI Fascial Closure Instrument [Karl Storz GmbH, Germany]). In our experience, we find that over time, the suture passer tip becomes blunt and its shaft will eventually start curving with repeated use. This makes subsequent insertion of suture passer a struggle with multiple attempts needed before a successful placement. Studies have shown that smaller diameter transfascial suture passers require a lower insertion force [12].

In view of this, we propose a novel alternative to suture passer in primary fascial closure. Intravenous cannulas are widely available in hospital settings. It is traditionally used for administration of fluids intravenously, invasive monitoring of blood pressure and blood sampling. It is also used for diagnostic and therapeutic aspiration of abscesses, peritoneal or pleural fluids. Here, we demonstrate a simple technique using a 14-gauge intravenous cannula (BD Angiocath™) as a novel alternative to transfascial suture passer.

**Fig. 2:**
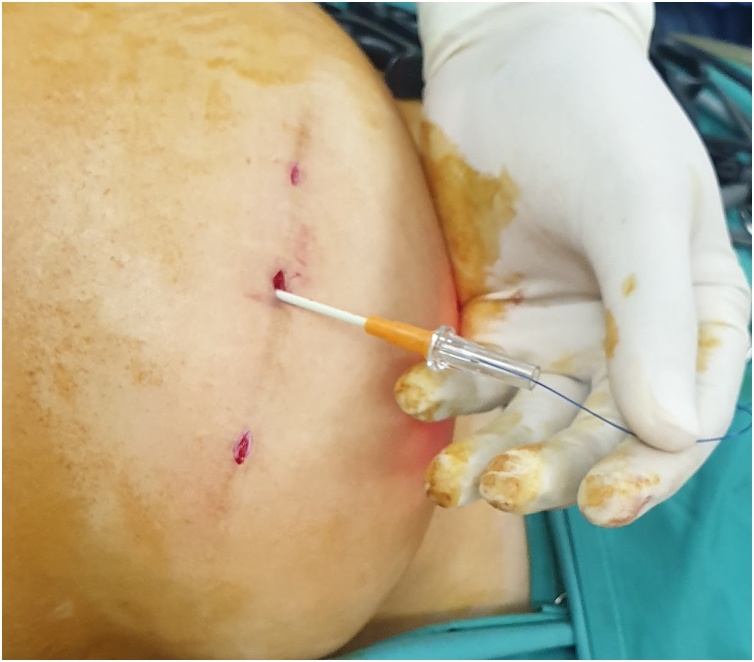
A large bore (14G) IV cannula is inserted through the abdominal wall fascia via a small skin nick incision.

**Fig. 3:**
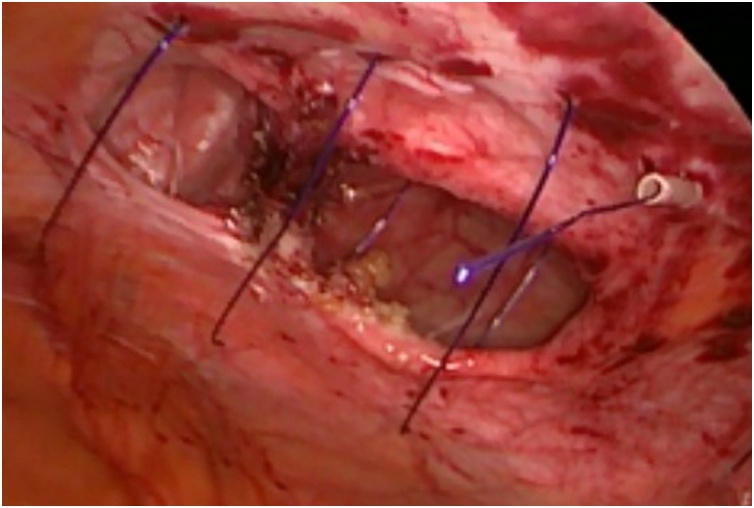
A non-absorbable suture (Prolene^®^ 0) is then inserted into the cannula.

**Fig. 4:**
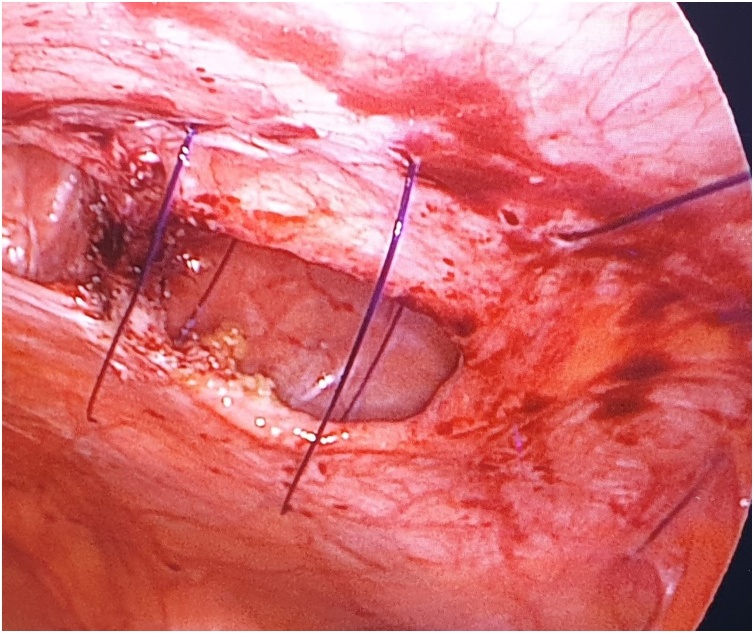
The IV cannula with stylet is withdrawn after a sufficient length of suture is inserted.

**Fig. 5:**
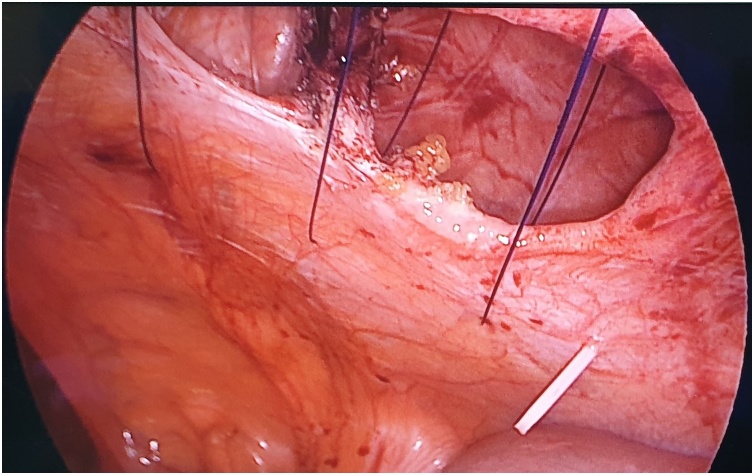
The same IV cannula is then directed through the same skin incision into the opposite edge of the fascial defect.

**Fig. 6:**
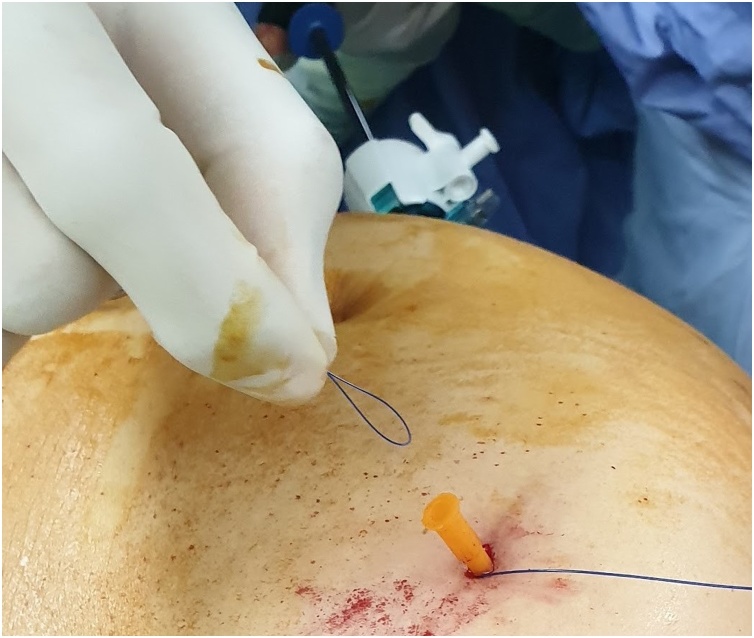
A loop of smaller-sized suture (Prolene^®^ 2/0) is passed into the lumen after the stylet is removed.

**Fig. 7:**
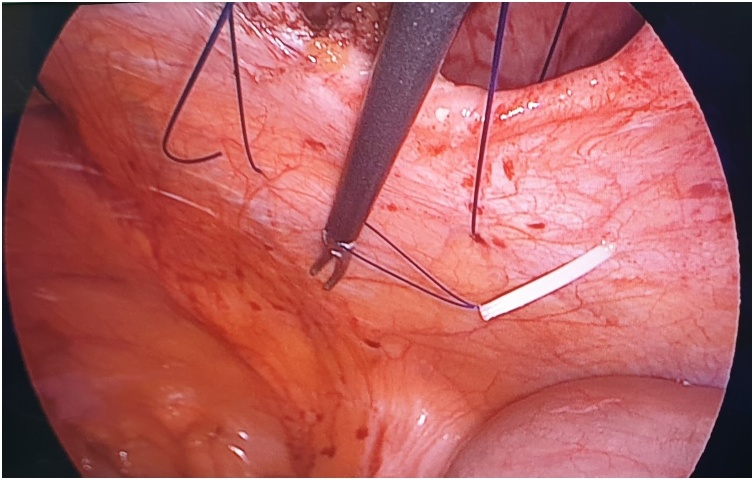
A laparoscopic grasper is passed through the loop to catch the suture placed earlier at the opposite fascial edge.

**Fig. 8:**
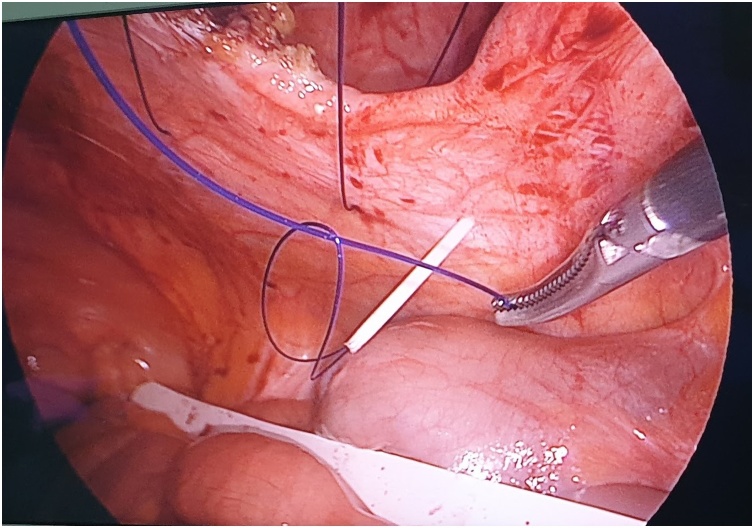
The suture is then pulled into the loop made of the smaller-sized suture.

**Fig. 9:**
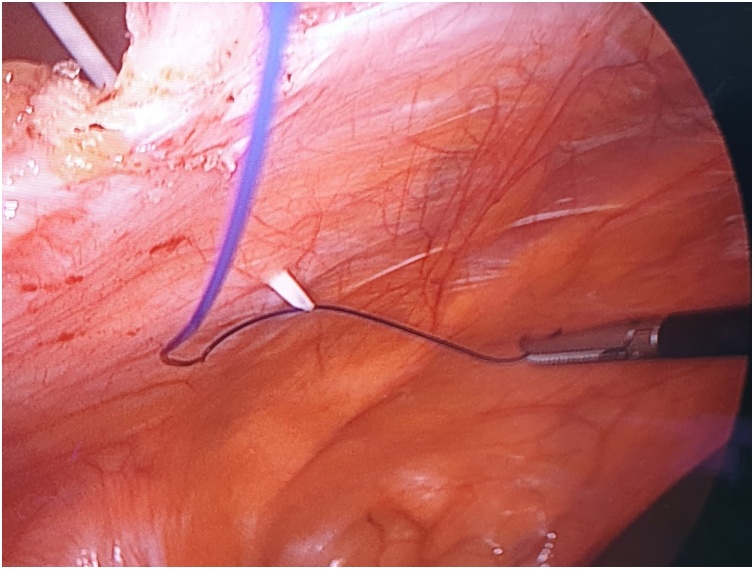
The suture is then fished into the IV cannula by pulling the loop.

**Fig. 10:**
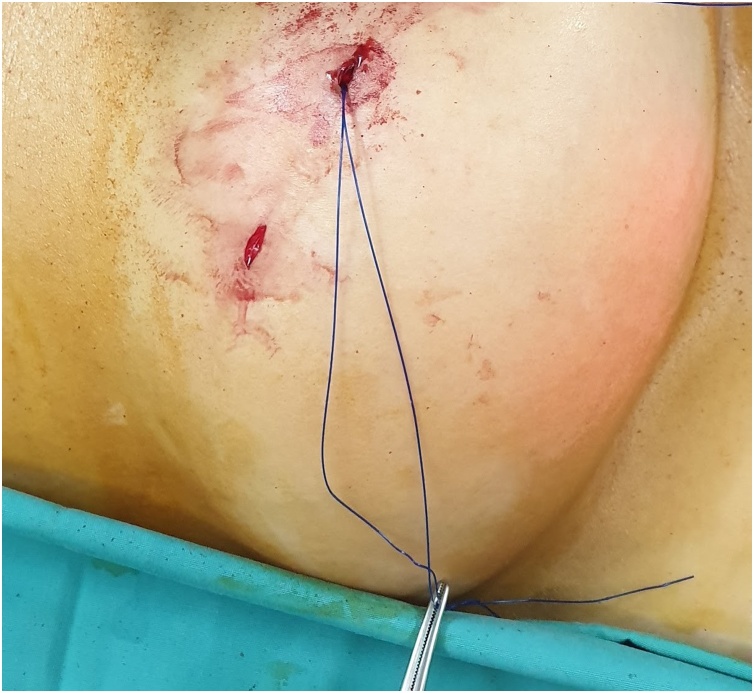
The IV cannula together with the fishing loop are removed once the fascial suture is thoroughly fished out externally.

**Fig. 11:**
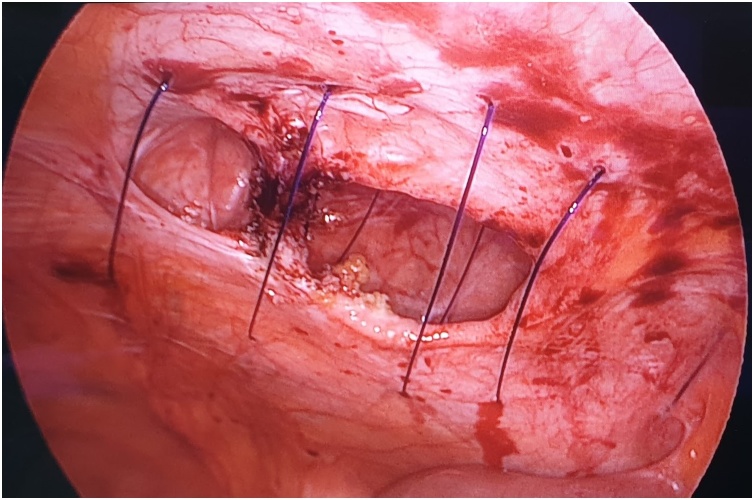
The completed fascial suture placement as seen from laparoscopic view.

**Fig. 12:**
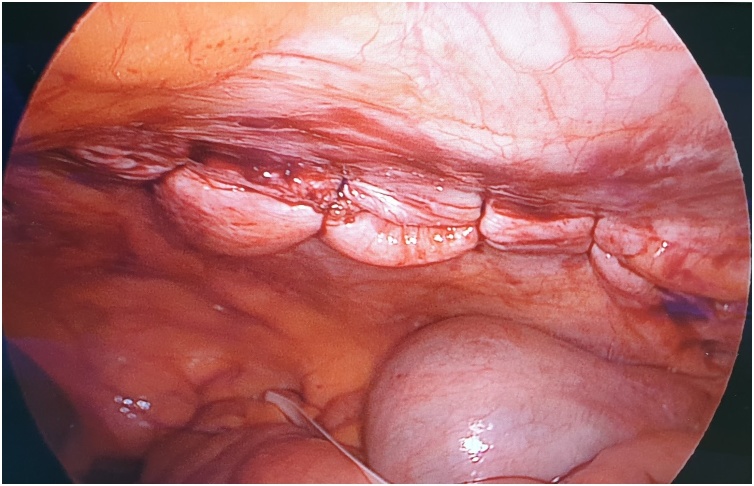
The fascial defect is opposed by tying the knots in the subcutaneous plane.

**Fig. 13:**
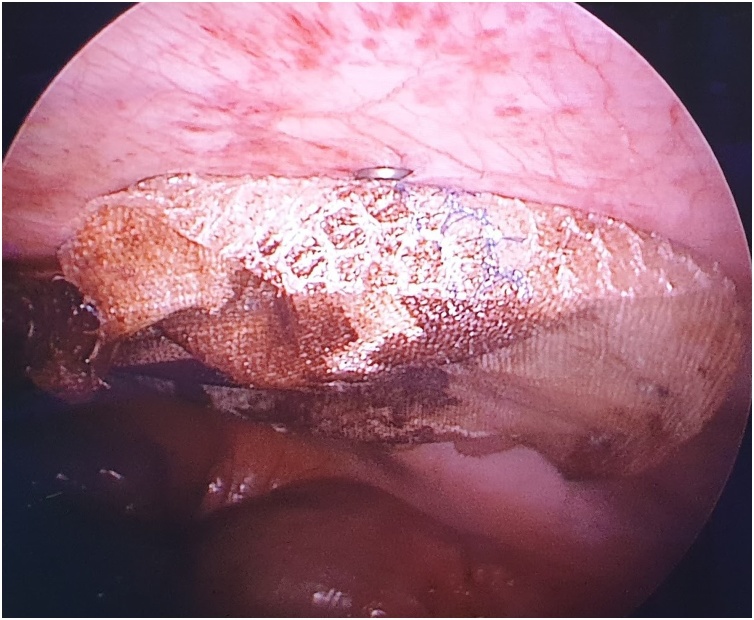
Intraperitoneal onlay mesh is then placed in the usual manner using laparoscopic tackers.

The first step is to make a skin nick incision to allow entry of the intravenous cannula. The intravenous cannula is then inserted vertically piercing one edge of the fascial defect under laparoscopic vision. With or without the stylet needle in place, a non-absorbable suture (Prolene^®^ 0) is introduced into the cannula (Fig. 2, Fig. 3). Once sufficient length of suture is inserted, the cannula is then withdrawn along with the stylet needle and redirected through the same skin incision into the opposite edge of the fascial defect (Fig. 4, Fig. 5). The stylet needle is then removed, and a non-absorbable suture of smaller diameter (Prolene^®^ 2/0) is then looped and inserted into the lumen of the cannula (Fig. 6). Using a laparoscopic grasper, the end of the earlier placed suture is then pulled into the loop and fished into the cannula by pulling the loop (Fig. 7, Fig. 8, Fig. 9). The suture is then pulled externally, and the cannula with the fishing loop is removed (Fig. 10). The steps described are repeated until the fascial suture placement is completed (Fig. 11). The fascial defect is then opposed by tying the knots in the subcutaneous plane (Fig. 12). A polypropylene coated mesh of adequate size is then anchored in place with tackers (Fig. 13).

The advantage of using an IV cannula instead of a suture passer is that they are widely available. Its single-use also eliminates the risk of transmissible diseases, and as it has a smaller diameter than suture passer, it requires a lower insertion force for successful placement [12]. This theoretically will reduce the risk of inadvertent abdominal visceral injury due to excessive insertion force. As an alternative economic option, the IV cannula does not pose any more harm than the suture passer but may offer extra benefits.

## 4 Conclusion

Primary fascial closure in laparoscopic IPOM repair may be beneficial. An intravenous cannula may be used as a more economical alternative to a transfascial suture passer. The advantage of using an IV cannula is that they are widely available, require a lower insertion force for successful placement, and zero risks of transmissible diseases from being single-use. This technique is easily reproducible and does not violate the principles of primary fascial defect closure in laparoscopic ventral hernia repair.

## Conflicts of interest

No conflict of interests.

## Funding

No source of funding.

## Ethical approval

The National University of Malaysia’s Ethics Committee has exempted the need for an ethical approval for any case report being written/ published.

## Consent

Written informed consent was obtained from the patient for publication of this case report and accompanying images.

## Author contribution

Study concepts: Nik Ritza Kosai, Mohamad Aznan Bin Shuhaili, Study design: Nik Ritza Kosai, Mohamad Aznan Bin Shuhaili.

Data acquisition: Lai Jun Han, Loo Guo Hou, Quality control of data and algorithms: Nik Ritza Kosai, Mohamad Aznan Bin Shuhaili, Data analysis and interpretation: Lai Jun Han, Loo Guo Hou, Statistical analysis: -Not applicable.

Manuscript preparation: Loo Guo Hou, Lai Jun Han.

Manuscript editing: Loo Guo Hou, Lai Jun Han.

Manuscript review: Nik Ritza Kosai, Mohamad Aznan Bin Shuhaili.

## Registration of research studies

Not applicable.

## Guarantor

Professor Dr Nik Ritza Kosai.

## Provenance and peer review

Not commissioned, externally peer-reviewed.
